# Retraction Note: The microRNA-325 inhibits hepatocellular carcinoma progression by targeting high mobility group box 1

**DOI:** 10.1186/s13000-018-0701-4

**Published:** 2018-05-02

**Authors:** Huifen Li, Weihua Huang, Rongcheng Luo

**Affiliations:** 1 Department of Chemotherapy, Zhongshan People’s Hospital, Zhongshan, 528400 Guangdong China; 2 TCM-Integrated Hospital, Southern Medical University, Cancer Center, NO.13 Shiliugang Road, Haizhu District, Guangzhou, 510315 Guangdong China

## Retraction

The editor has retracted this article [1] because it shows significant overlap with the following articles (amongst others) [2, 3]. None of the authors responded to correspondence regarding this retraction.
